# Use of Real-World Evidence in Regulatory Decisions for Traditional Chinese Medicine: Current Status and Future Directions

**DOI:** 10.1007/s43441-023-00588-0

**Published:** 2023-11-23

**Authors:** Pei Li, Su Wang, Yuwen Chen

**Affiliations:** 1 School of Business Administration, Shenyang Pharmaceutical University, Shenyang, 110016 People’s Republic of China; 2 Drug Regulatory Research Base of NMPA - Research Institute of Drug Regulatory Science, Shenyang Pharmaceutical University, Shenyang, 110016 People’s Republic of China

**Keywords:** Traditional Chinese Medicine (TCM), Real-world evidence (RWE), Regulatory decisions, Current status and future directions, Evidence-based regulatory framework

## Abstract

Traditional Chinese medicine (TCM) is a valuable resource unique to China with a long history of human use and clinical practice, which can be analyzed to generate real-world evidence (RWE). The Chinese government has been actively promoting regulatory reform that is in line with the characteristics of TCM, optimizing the clinical evidence system for TCM, and exploring the important role of RWE in supporting the development of new drugs and regulatory decision-making for TCM. This article aims to provide a comprehensive review of the use of RWE in regulatory decisions for TCM. Based on the characteristics of TCM, this study focuses on the application scenarios, challenges, and opportunities of RWE in TCM. And some suggestions are put forward to promote the wider application of RWE in TCM development and supervision.

## Introduction

In recent years, the value of real-world evidence (RWE) in the field of pharmaceutical product development has gained significant attention worldwide. National and regional drug regulatory agencies, such as the FDA, EMA, and MHRA, have extensively explored the utilization of RWE to support regulatory decision-making for chemical drugs and biologics. China, renowned for its unique resource of traditional Chinese medicine (TCM), presents an intriguing opportunity for the application of RWE. With a history spanning thousands of years, TCM has accumulated extensive empirical knowledge and a substantial repository of data from its use in preventing and treating various diseases [1]. Furthermore, TCM has been increasingly embraced globally as a complementary and alternative healthcare option [1, 2].

However, the regulatory landscape for TCM can be complex, particularly in evaluating its safety and efficacy [3]. Traditional randomized controlled trials (RCTs), considered the gold standard for assessing drug efficacy and safety, may not always be feasible or suitable for TCM products due to their distinctive characteristics. Consequently, there is growing interest in leveraging RWE to complement conventional clinical trials and gain insights into the real-world effectiveness of TCM.

The systematic use of RWE to support drug regulatory decision-making is still in its infancy but rapidly developing in China [4]. The application of RWE in TCM regulation is gradually gaining traction, reflecting the recognition of its potential benefits. This study focuses on exploring the application scenarios, challenges, and opportunities associated with the use of RWE in TCM, considering its unique characteristics. By providing international perspectives on the research and regulatory paradigm of RWE in China, this study contributes to advancing the understanding and implementation of RWE in the field of TCM.

## Introduction to TCM and Its Regulatory Landscape in China

TCM encompasses a wide range of items, Chinese patented medicines are the focus of this article. TCM products are often composed of multiple natural ingredients, such as herbs, minerals, and animal products, and their manufacturing process and quality control standards can differ from those of conventional drugs [2]. Furthermore, evaluating the safety and efficacy of TCM can be challenging due to its complex formulations and individualized approach to treatment.

In China, the supervision and administration on TCM is as strict as that of chemical drugs and biological products [5]. TCM refers to medicinal substances and their preparations used under the guidance of traditional Chinese medical theory, according to the Provision for Drug Registration, which provides practical guidelines for the registration of drugs [6]. Drugs are regulated by the National Medical Products Administration (NMPA) and TCM is controlled by Department of TCM and Ethnic Medicine under Department of Drug Registration Administration (Department of Supervision and Administration of Traditional Chinese Medicine and Ethnic Medicines), which has developed a separate regulatory system for TCM [7]. Registration of TCM is subject to strict technical evaluation on its safety, efficacy, and quality, which is conducted by the Center for Drug Evaluation (CDE), NMPA [8].

RCTs are considered the gold standard for evaluating the safety and efficacy of new drugs, including new TCM drugs. However, there are several limitations to using RCTs to evaluate TCM products that are based on a holistic approach to healthcare and individualized treatment. The Chinese government has been actively promoting regulatory reform for TCM. In October 2019, the CPC Central Committee and The State Council issued the Opinions on Promoting the Inheritance and Innovation of Traditional Chinese Medicine, which proposed to “accelerate the establishment of a TCM registration and evaluation evidence system combining TCM theory, human use experience and clinical trials” [9]. Human use experience is derived mainly from real-world studies (RWS), which generate RWE. Policies and measures [10–12] require the establishment of a TCM characteristic evaluation evidence system, and actively explore the establishment of a RWS evidence system for TCM. As a new tool and method for clinical research on TCM, RWE has been increasingly valued by regulatory agencies. Regulatory agencies have actively explored the introduction of RWE into TCM registration management. In 2020, NMPA released the related guidelines to promote the use of RWE in the development and registration of medical products, including TCM products. It is encouraged to use RWE for the evaluation of TCM efficacy. It also means that RWE can be used as part of the evidence for supporting the listing of products [13–15].

## RWE Application Scenarios in the Development and Regulation of TCM

### Exploring New Approaches for Clinical Research and Development of Well-known Prescriptions/Formulas from Experience of the TCM Practitioner

The research and development mode based on “clinical experience prescription/ TCM preparations in medical institutions-innovative Chinese medicines” has attracted widespread attention from the industry [16–19]. TCM compound preparations, especially TCM preparations in medical institutions, are the main source of the development of new TCM drugs [18, 19]. A research and development strategy that combines RWS with randomized clinical trials was introduced and the use of RWS to transform TCM preparations into new TCM drugs was encouraged by regulators. Observational studies (both retrospective and prospective) could be explored instead of phase I and/or II clinical trials to initially explore clinical efficacy and safety. Then, the effectiveness of TCM was further confirmed by RCTs or PCTs (Pragmatic Clinical Trials) or scientifically sufficient observational studies, providing supporting evidence for the registration and marketing of the products [13, 20].

There are some research and cases on the application of RWS in the research and development of new TCM drugs [21]. For TCM preparations in medical institutions, “Baiyunshan Toujie Quwen Granule (Pneumonia No.1)” has been registered and approved by Guangdong Provincial Food and Drug Administration in 2020, and it is the first preparation in medical institutions approved based on RWE in China [22, 23]. A retrospective observational study of Qingfei Paidu granules (treatment for COVID-19), with 3715 cases from more than 60 medical institutions in 28 provinces, from human use empirical evidence to support NDA approval [24]. In June 2023, Antiwei granules (treatment for cold), whose recipe was derived from Zhang Zhongjing’s “On Febrile Diseases” in the Han Dynasty, was approved by NMPA to move directly to Phase III studies due to prior human use experience [25].

### Use of RWE in Support of Labeling Changes for an Approved TCM

It has been proposed that RWE can be an important consideration in supporting label expansions, such as (1) Add or modify an indication; (2) Change in dose, dose regimen, or route of administration; (3) Use in a new population; (4) Add the results of the effectiveness comparative study; (5) Add safety information; (6) Others [13, 26]. Currently, reasonable scenarios for using RWE to support labeling changes include [27–30] (1) Safety contents are missing in the instructions of some listed TCM products, it is necessary to use RWE to add safety information to existing drug labels. (2) Off-label use occurs frequently, with an urgent need for relevant data to support the efficacy and safety. (3) Based on the large amount of real-world data (RWD) accumulated in the process of diagnosis and treatment, it is explored and found that the target drug also has potential clinical efficacy for the relevant complications of the main indications. (4) In the process of using the drugs in accordance with the instructions, discovering the existence of additional therapeutic effects (including adverse reactions/side effects that can also potentially benefit the patients), thus providing reference directions and bases for subsequent research. (5) Through the integration of a large number of RWD, it is found that the applicability of the relevant drugs can be further broadened to children and other special populations. (6) The range of functional indications in some listed TCM products is broad or the expression without clear indications, appropriate indications can be selected within the range of indications in the labels to carry out RWS, so as to provide efficacy and safety data for the modification of indications.

### Being Used as Part of a Post-marketing Requirement to Support a Regulatory Decision

RWE can also provide valuable information on rare events and adverse effects that may not be captured in RCTs. TCM treatments often involve the use of multiple herbs and are based on complex diagnostic criteria, making it difficult to predict all possible interactions and adverse events. RWE can provide insights into the safety of TCM treatments in real-world settings, including the occurrence of rare or unexpected events. Post-marketing requirements, such as “The applicant is required to strengthen the post-marketing effectiveness and safety research,” are included in the approval document [31]. For example, retrospective observational study of Xuanfei Baidu Granules was used as part of a post-marketing requirement to support a regulatory decision [32]. A new TCM drug, approved with a small number of cases for the treatment of leukemia, was required to conduct clinical research after marketing to fully prove its safety and effectiveness [33]. It should be noted that for the new TCM drugs, RWS can be carried out on their own in the pre-market clinical practice of TCM, which could be an alternative to post-market RWS [34].

### Use of RWE in Re-evaluation of Chinese Patent Medicine

Due to historical reasons, most of the Chinese patent medicine products currently on the market have not undergone rigorous clinical studies, especially the varieties approved by local authorities, which account for a considerable proportion of the existing Chinese patent medicine products [35]. Overall, the research foundation is relatively weak, and it is necessary to carry out re-evaluation work. Real-world data (RWD) can be fully utilized to conduct effectiveness and safety re-evaluations. The drug regulatory agency formulated work plan on the Chinese patent medicine re-evaluation (2009), especially for TCM injections [36, 37]. At the same time, some scholars proposed using real-world studies to assist in the re-evaluation of TCM injections [38]. As of early 2016, the coverage of real-world TCM injections has been limited. In April 2016, the RWS results of Xiyanping injection, Shuxuetong injection, Shenqi Fuzheng Injection, and other TCM injections were reported at the “The Fourth Annual Academic Meeting of the Professional Committee of Post-Listing Reevaluation of Chinese Medicine and the Summit Forum on Promoting the Development of Chinese Medicine Industry After Listing.” Since then, studies on the efficacy and safety of TCM injections have gradually become a very influential frontier hot spot [39–42]. In October 2017, the government issued a policy document [43], which clearly proposed to re-evaluate injection drugs that are already on the market (including TCM injections), and strive to complete it in about 5 to 10 years. Re-evaluation of Chinese patent medicine, especially TCM injections, is an important work that NMPA continuously promotes, and RWS can effectively promote the implementation and development of this work.

### The Use of RWS in Optimization Design of Clinical Research

RCTs strictly control the test inclusion, exclusion criteria, and other conditions. RWS can generate a large number of valuable RWE, which can provide a valid reference point for inclusion and exclusion criteria, parameters for sample size estimation, and determination of non-inferiority thresholds [13, 44].

For rare or serious diseases that are seriously life-threatening and lack effective treatment means, RCTs may not be carried out due to small patient population or ethical reasons, so RWS can be used as an external controlled single-arm study [45]. RWD also can be used to solve the problem of difficult recruitment of control group in TCM RCTs, that is, RWD is used as the control group in TCM RCTs. This kind of design can effectively solve the problem of difficulty in the enrollment of Chinese and western control group patients in TCM RCT, and can provide strong evidence to evaluate the efficacy of TCM [46].

### Others Such as Elucidate and Enrich TCM Theory

TCM has accumulated rich experience in disease prevention and treatment, and formed the theory. We need to further deepen the understanding of the clinical value and characteristic advantages of TCM, and be able to prove it with research evidence. For example, in clinical practice, it is found that a therapy is more effective or has more adverse reactions for a certain kind of patients, or that the compatibility of a certain drug is more significant [44, 47]. It is a good way to elucidate and enrich TCM theory with RWS.

## Challenges and Opportunities for Using RWE in TCM Regulation

### Challenges for Using RWE in TCM Regulation

#### The Complexity and Diversity of TCM Terminology

The diversity and complexity of TCM terminology have brought some obstacles to the quantification of TCM clinical information in the RWS. The standardization of TCM terminology needs further study, and the application of standardized results is insufficient and disjointed from clinical and scientific research [48, 49]. Therefore, how to standardize TCM terminology to meet the needs of information extraction in the RWS is an urgent problem to be solved.

#### Difficulties in Data Collection and Integration

Challenges of data collection and integration in TCM RWS[50–56]: (1) The data distribution of hospital medical records, ancient books of traditional Chinese medicine monographs, experience of famous old Chinese medicine practitioners, and other data are scattered. (2) Data standards are not uniform when establishing databases. (3) There is also the phenomenon of “data silos” (defined simply as isolated systems with limited external connectivity) in data sharing. (4) No unified universal data model nor semantic standard exists in China. (5) Patient engagement and data privacy protections are inadequate. (6) RWS, especially retrospective RWS, may have limitations in tracking long-term outcomes and assessing the durability of TCM interactions, such as high loss rate, incomplete, and inaccurate follow-up content.

Thus, it is difficult to avoid the problems of excessive data volume, unstructured data, data missing, and strong data heterogeneity in RWS.

#### Difficulties in the Quality Control of TCM RWS

TCM clinical practice has its own particularities, such as the complicated diagnosis and treatment modality of doctors based on syndrome differentiation and complex intervention of internal and external treatment, which all determine the particularity and difficulty of TCM RWS. There are few published reports of RWS on TCM that clearly specific quality control measures, which influenced the results of RWS reliability [57, 58].

#### Confounding and Bias Control Difficulties

Non-randomization of RWS inevitably results in confounding and bias, which may distort the true association between exposure and outcome. Confounding and bias control difficulties in real-world TCM studies arise from the complexity of interventions, heterogeneity of practice, lack of standardized protocols, patient self-selection bias, and limited control groups. Compared with chemical drugs and biologics, TCM may have more confounding and bias in RWS, such as the confounding and bias introduced in the complicated process of syndrome differentiation and treatment (syndrome, syndrome type, syndrome differentiation, drug use, etc.)[58, 59].

#### Limited Evidence Hierarchy

Compared to RCTs, RWE in TCM research is often considered lower in the evidence hierarchy [60, 61]. This can lead to challenges in gaining acceptance and recognition from regulatory bodies, healthcare professionals, and researchers. An important ‘challenge’ that is the risk that evidence generated using RWD is flawed, may leading to people being harmed. TCM RWS in China mainly focused on the analysis of the efficacy of Chinese medicine injections and big data-based clinical medication rule analysis, with the main research type of observational study [39]. RWE application and scientific nature need further improvement.

### Future Directions and Opportunities for Using RWE in TCM Regulation

Given the potential benefits and challenges of using RWE in TCM regulation, there are several future directions and opportunities that can be pursued to further advance the field. These include the following:

#### Harmonizing and Standardizing TCM Terminology

The quantification and standardization of TCM clinical information is the foundation of TCM clinical information management. Studies have shown that TCM terminology (such as real-world clinical diseases of symptoms) will be the focus of future standardization research [62]. It is recommended to create a standard library of terminology specific to clinical research and to establish a research pathway for standardization of TCM terminology based on real-world research needs.

#### Data Collection and Integration

The collection and integration of TCM data is an important step of RWS. The inter-regional/inter-institutional information system connectivity, data standardization, and integrated data management should be promoted. The construction of TCM clinical research big data platforms is a realization method. At present, there are some shortcomings of the existing platforms. Regulatory Agencies are building digital platforms for real-world Chinese medicine research, accelerating the drafting of policy documents such as real-world Chinese medicine research guidelines and data collection standards [63, 64].

There is also proposed to innovate data collection models and explore diversified follow-up methods. For example, Guangzhou Greater Bay Area Real World Research Center of Traditional Chinese Medicine is the first research institution in China focusing on RWS of TCM based on family doctors and is exploring a new cooperation model of “inside and outside hospitals” for RWS, which is more conducive to obtain complete data, especially follow-up data [65]. The sponsors and this research institution should be responsible for making maintaining this data.

Patient engagement and data privacy are important considerations in the use of RWE in TCM regulation. Data privacy should be safeguarded through the use of secure data storage and transmission methods. In addition, it is suggested improving traceability to source data for transparency.

#### Data Quality Management and Standardization

The reliability of evidence is directly dependent on the quality of RWS conducted on TCM. When it comes to drug registration materials, it is essential to establish clear quality requirements for RWE. These requirements encompass various aspects, including basic prerequisites, organizational management, key pharmaceutical information, scientific research, risk management, ethical compliance, and study implementation [66]. To ensure standardized and high-quality RWS on TCM, it is crucial for regulatory authorities, applicants, research institutions, and researchers to collaborate to establish a robust research quality management system that aligns with the clinical characteristics of TCM.

#### Confounding and Bias Control

The more complex the research environment and data sources, the more complex the data processing and analysis techniques. On the one hand, relying on the TCM Regulatory Science Action Plan, there is an urgent need for interpreting RWS results with caution, and exploring methods to improve their scientific validity, reliability, and methodologies to control various biases. On the other hand, it is necessary to establish standardized and rigorous research design and statistical analysis, which puts higher requirements on sponsors and statistical experts.

#### Understanding and Rational Use of RWE

As RWE sources and analytics continue to advance, there has been new opportunities for using RWE in TCM regulation, such as RWE application scenarios mentioned above. Only RWD that meets the applicability, which is evaluated primarily by data relevance and reliability, can produce RWE. Compared with RCTs, RWE is not a lowering of standards; it can complement the evidence provided by traditional clinical trials to form a complete and rigorous evidence chain, rather than replace it.

In addition, education and training programs should also be developed to increase awareness and understanding of RWE among TCM practitioners, researchers, and regulators.

## Conclusion

TCM has accumulated rich experience in human use and a massive amount of data, which can be analyzed to generate RWE. The use of RWE in regulatory decisions for TCM in China has great potential to support TCM development and approval. In recent years, there has been growing interest in the use of RWE in TCM regulation. While there are challenges and limitations associated with using RWE in TCM regulation, efforts should be made to standardize TCM diagnosis and treatment, develop robust RWE sources, integrate RWE and RCTs, promote collaboration among stakeholders, and provide education and training to increase awareness and understanding of RWE in TCM. By doing so, we can further advance the use of RWE in TCM regulation and improve the health outcomes of patients who use TCM treatments.

Noteworthy is: This is a review of existing research and regulations related to the incorporation of RWE to support new guidelines in the conduct of RCTs with TCM.
